# Association of air pollution and homocysteine with global DNA methylation: A population-based study from North India

**DOI:** 10.1371/journal.pone.0260860

**Published:** 2021-12-02

**Authors:** Suniti Yadav, Imnameren Longkumer, Priyanka Rani Garg, Shipra Joshi, Sunanda Rajkumari, Naorem Kiranmala Devi, Kallur Nava Saraswathy

**Affiliations:** 1 Laboratory of Biochemical and Molecular Anthropology, Department of Anthropology, University of Delhi, Delhi, India; 2 MAMTA Health Institute for Mother and Child, New Delhi, India; 3 Manbhum Ananda Ashram Nityananda Trust-MANT, Kolkata, West Bengal, India; Texas Tech University, UNITED STATES

## Abstract

**Background:**

Anthropogenic air pollution has been implicated in aberrant changes of DNA methylation and homocysteine increase (>15μM/L). Folate (<3 ng/mL) and vitamin B_12_ (<220 pg/mL) deficiencies also reduce global DNA methylation via homocysteine increase. Although B-vitamin supplements can attenuate epigenetic effects of air pollution but such understanding in population-specific studies are lacking. Hence, the present study aims to understand the role of air pollution, homocysteine, and nutritional deficiencies on methylation.

**Methods:**

We examined cross-sectionally, homocysteine, folate, vitamin B_12_ (chemiluminescence) and global DNA methylation (colorimetric ELISA Assay) among 274 and 270 individuals from low- and high- polluted areas, respectively, from a single Mendelian population. Global DNA methylation results were obtained on 254 and 258 samples from low- and high- polluted areas, respectively.

**Results:**

Significant decline in median global DNA methylation was seen as a result of air pollution [high-0.84 (0.37–1.97) vs. low-0.96 (0.45–2.75), p = 0.01]. High homocysteine in combination with air pollution significantly reduced global DNA methylation [high-0.71 (0.34–1.90) vs. low-0.93 (0.45–3.00), p = 0.003]. Folate deficient individuals in high polluted areas [high-0.70 (0.37–1.29) vs. low-1.21 (0.45–3.65)] showed significantly reduced global methylation levels (p = 0.007). In low polluted areas, despite folate deficiency, if normal vitamin B_12_ levels were maintained, global DNA methylation levels improved significantly [2.03 (0.60–5.24), p = 0.007]. Conversely, in high polluted areas despite vitamin B_12_ deficiency, if normal folate status was maintained, global DNA methylation status improved significantly [0.91 (0.36–1.63)] compared to vitamin B_12_ normal individuals [0.54 (0.26–1.13), p = 0.04].

**Conclusions:**

High homocysteine may aggravate the effects of air pollution on DNA methylation. Vitamin B_12_ in low-polluted and folate in high-polluted areas may be strong determinants for changes in DNA methylation levels. The effect of air pollution on methylation levels may be reduced through inclusion of dietary or supplemented B-vitamins. This may serve as public level approach in natural settings to prevent metabolic adversities at community level.

## Introduction

Atmospheric pollution as a consequence of rising anthropogenic activities has long been associated to detrimental effects on health [1–4]. According to WHO, 92% of the world population resides in places where air quality exceeds the normal limits and ambient air pollution exposure has been reported to be particularly high in low- and middle-income countries [5]. Global human mortality attributed to ambient air pollution has been reported to be approximately 3 million per year, of which highest number of the deaths are from South-East Asia and Western Pacific regions [5, 6].

The adverse effects of long term exposure to PM_2.5_ (10 μg/m^3^ annual mean), PM_10_ (20 μg/m^3^ annual mean) (particulates with aerodynamic diameter <2.5μm and <10μm, respectively) on human health in terms of cardio-respiratory adversities, cancer and neurological effects have been widely covered in epidemiological studies [6–12]. Elevated localized PM_2.5_ levels are specifically reported to be much higher near the highway owing to vehicular emissions compared to the far-field regions [13–16]. These fine emission particles get deposited in the lower respiratory tract and trigger local and systemic inflammation [17] which in turn may set off a cascade of metabolic reactions. One of the prominent widely studied inflammatory marker, homocysteine, is also linked to air pollution in numerous studies, possibly through oxidative stress [18, 19]. However, micronutrient deficiencies (B vitamins including folate, vitamin B_12_ and vitamin B_6_) are also well-known reasons for homocysteine increase i.e. hyperhomocysteinemia [19, 20]. High circulating levels of homocysteine (>15umol/L) are considered as an independent risk for cardiovascular diseases including atherosclerosis, endothelial dysfunction, systemic inflammation, and even stroke [21].

Recent advances have focused on metabolic adversities such as hypertension [22], dyslipidemia [23], insulin resistance [24], cardiovascular diseases [25], pulmonary dysfunction [26] caused by air pollution through changes in gene expression via alteration in the epigenome and DNA damage. Thus, it may serve as an intermediate phenotype in the exposure-health effect continuum [27] that can be explored through environmental epigenetics. DNA methylation may act as one of the mediators of epigenetic phenomenon and may have a direct effect on the disease phenotype [28]. Hence, any alterations in DNA methylation may be indicative of disease onset or progression [25]. DNA methylation is a dynamic phenomenon that gets rapidly altered in peripheral leukocytes due to exposure to ambient air pollutants [17, 27, 29, 30]. Global DNA methylation is a quantitative estimate of total level of 5-methyl cytosine in the genome [31]. Differential DNA methylation patterns across genome have been reported in pollutant exposure but majority of studies have reported hypomethylation to be associated with an increased air pollution exposure [27, 32–38]. On the other hand, homocysteine is a key molecule in the one-carbon metabolic pathway that governs the supply of free methyl groups for DNA methylation. Global DNA hypomethylation is primarily a result of unavailability of methyl groups which could possibly be a consequence of disturbances in one carbon metabolic pathway such as micronutrient deficiencies and genetic polymorphisms involved in the pathway. This consequential hypomethylation is likely to be aggravated by ambient air pollution.

In recent years, India has been the fastest growing stable economy of the world owing to massive contribution by industrialization and urbanization [39]. Due to rapid increase in anthropogenic activities − industrialization and connectivity by road transportation, there is a visible amplification in air pollution specifically in the National Capital Region (NCR) of Delhi. The areas on/near to National highways are therefore expected to be more polluted compared to far off areas from the national highways.

Thus, the present study is an attempt to understand the effect of air pollution and homocysteine on global DNA methylation independently and also in light of micronutrient deficiencies (vitamin B_12_ and folate) among individuals from a single population.

## Methods

### Study population and study design

The present cross-sectional study was a part of a major research project funded by Department of Biotechnology, Government of India. Door to door household surveys were done and members belonging to a single community residing in two different geographical areas (low and high polluted) were identified. Individuals belonging to the same community, both males and females in the age group between 35–65 years were recruited for the study. Exclusion criteria were presence of any severe morbidity such as CVDs or infectious diseases such as tuberculosis, HIV or pregnant/lactating women and bed ridden. Individuals belonging to different communities, ethnicities and regions were also excluded from the present study. As the project involved the collection of blood samples and genetic analysis, the major criteria for the recruitment of participants was avoidance of individuals related upto first cousins, identified from detailed genealogies. The study population was largely lacto-vegetarian with only 9% of the population consuming egg/non-vegetarian food occasionally. All the data were collected after obtaining prior informed written consent from each participant.

A total of 823 individuals from low polluted and 811 individuals from high polluted areas of either sex in the age group 35–65 years were recruited for the study. For global DNA methylation assay, every third individual in both high and low polluted areas was selected, resulting into 274 samples from low polluted and 270 samples from high polluted areas. The study was approved by Department Ethics Committee, Department of Anthropology, University of Delhi, Delhi. All methods were conducted in accordance with the Helsinki Declaration and ICMR’s National ethical guidelines for biomedical and health research involving human participants.

### Unit of study

Two areas i.e. low polluted and high polluted from district Palwal in Haryana state, located 56km from National capital region of Delhi were considered for the present study. Villages in this district were segregated on the basis of their distance from National Highway (NH)-2. A more strict criteria than that proposed by Nagendra et al., [13] and secondary literature were chosen for the present study to categorize villages into low and high polluted areas based on location of brick quarries [14] and vehicular count. The villages that were located at a minimum distance of 10km from NH-2, no main road in close proximity (upto 5km), less than two *pucca* roads (black topped road) in the village, no factories/brick quarries (upto 5km), vehicular count **<**100 vehicles per day, distance of residential area from the main road >1km, were categorised into low polluted areas. On the other hand, the villages located on/in proximity to NH-2 (within 1km radius), having more than two *pucca* roads in each village, factories/brick quarries (within 2km radius), vehicular count >10,000 for 24 hrs, were categorized into high polluted areas. Nine villages were selected from low polluted areas in Block Hathin whereas six villages from high polluted areas in Block Hodal were covered.

### Fieldwork and data collection

Fieldwork for total project was conducted over a period of two years by a team of four individuals. To account for seasonal variability in air pollution, the team ensured that equal number of study participants were recruited from both low and high polluted areas during a particular stretch of fieldwork. One of the limitations of the study is the absence of assessment of primary level of air pollutants and to overcome this, the study in the two areas was equally distributed across the year accounting for a balance in the effect of air pollution and seasonal variations. The data on personal identification, genealogies, demographic and lifestyle variables as described above were collected from all the study participants.

### Blood sample collection

Overnight fasting intravenous blood sample (5mL) was collected into evacuated tubes with and without EDTA (2.5mL each) from every participant and was transported on ice-box to the Laboratory of Biochemical and Molecular Anthropology, Department of Anthropology, University of Delhi for processing within two-three hours of collection. Plasma and serum were separated from evacuated tubes with EDTA and without EDTA, respectively and stored after coding the tubes at -80°C freezers until further analysis. DNA was extracted from the evacuated tubes with EDTA after separation of plasma using salting-out method [40]. All biochemical assays were carried out by the same team on the same equipment throughout the study. Prior to each assay, quality controls were run and a minimum of 10% of the total samples were repeated for analysis to check for intra- and inter-assay variations. The intra- and inter-assay coefficients of variation was <10%.

### Biochemical analysis

Serum folate, serum vitamin B_12_ and plasma homocysteine levels were assessed by chemiluminescence technique by Immulite2000 (Siemens Healthineers, Henkestr, Erlangen, Germany).

### Classification of biochemical variables

Cut-off values for homocysteine, folate and vitamin B_12_ levels were considered according to Refsum et al. [41]. Hyperhomocysteinemia: defined as homocysteine >15 μM/L, Folate deficiency: defined as folate <3 ng/mL, Vitamin B_12_ deficiency: defined as vitamin B_12_ <220 pg/mL.

### Global DNA methylation assay

Global DNA methylation levels were analyzed using ELISA based colorimetric technique as per manufacturer’s instructions (MethylFlash™ Methylated DNA Quantification Kit-Colorimetric, Epigentek Group Inc., New York, NY, USA, cat. no. P-1034–96). To avoid handling variations, this assay was performed by a single individual in duplicates with inter- and intra-assay variations <10%. Mean of the two values for each sample was taken for further statistical analysis. The result for global DNA methylation assay could be obtained on 254 samples from low polluted and 258 samples from high polluted areas for some technical reasons such as inter-assay variation >10%.

### Variability in data

All biochemical assays were performed with accuracy. However, variability in final data for biochemical assays was noted. This was due to technical reasons such as higher values beyond range for biochemical tests (homocysteine, folate, vitamin B_12_) or inter/intra-assay variation >10%. The tests for these assays were repeated and if the similar results were obtained, the samples were not included in the subgroup analysis.

### Statistical analysis

Data were entered in MS-Excel and checked twice by two individuals separately to ensure integrity, non-duplication and accuracy. Data were reported as mean ± standard deviation for continuous variables and number (percentage) for categorical variables. Difference between categorical variables was tested using χ^2^ test. The normality test for continuous variables was done and median values were considered where the distribution was not seen normal. Differences between the continuous variables were tested using student t-test. Median (interquartile range i.e., IQR) values for global DNA methylation, folate and vitamin B_12_ were used and median (IQR) values were compared by Mann-Whitney test for two groups.

Effect sizes were calculated for each group for which median values were compared. Effect size (r) was calculated as z-statistic value divided by √N. Multiple testing correction for p-values was performed where two or more tests were performed. For corrected p-value, actual p-values were corrected for the number of tests performed. Collinearity diagnostics were checked before conducting regression analysis. Eigen values (not close to zero) and condition index (less than 15) were considered while checking for multi-collinearity. Stepwise multiple linear regression analysis and backward regression were done to identify determinants of global methylation. The models included the following variables: age, sex, smoking, alcohol consumption, education, occupation, homocysteine, folate, vitamin B_12_ and air pollution. Statistical analysis was performed using SPSS version 16.0 for windows (SPSS Inc., Chicago, Illinois, USA) and p-value ≤0.05 was considered as statistically significant.

## Results

The present study attempts to understand the relation between air pollution, homocysteine, micronutrient deficiency (folate and vitamin B_12_) and global DNA methylation. It is part of a major funded research project where individuals from a single Mendelian population residing in two different environmental settings i.e. low polluted (823) and high polluted (811) were recruited. Data on demographic and lifestyle variables along with fasting blood samples were collected from all the participants. Biochemical assay (homocysteine, folate and vitamin B_12_) was performed on all the samples. For the present study, every third individual from the respective area was selected randomly for global DNA methylation assay due to funding issues.

### Study population and characteristics

The presently studied groups did not differ significantly with respect to their demographic (age, sex) and lifestyle (education, occupation, smoking status, alcohol consumption and diet) variables with respect to air pollution (Table 1). Individuals with hyperhomocysteinemia and vitamin B_12_ deficiency were significantly higher in high polluted areas whereas individuals with folate deficiency were significantly higher in low polluted areas. Median (IQR) levels of homocysteine and folate were significantly higher in high polluted areas compared to low polluted areas. No difference was observed in the median (IQR) levels of vitamin B_12_ between high and low polluted areas (S1 Table).

**Table 1:** Distribution of demographic and lifestyle variables among individuals in low and high polluted areas.

Variables		Low polluted (N = 254)	High polluted (N = 259)	χ^2^ p-value
Age (years)* (Mean±SD)		47.9±8.9	48.2±10.2	0.72*
**Sex**	Females	173 (68.1%)	187 (72.2%)	0.31
	Males	81(31.9%)	72 (27.8%)	
**Education**	Literates	143 (56.3%)	126 (50.6%)	0.20
	Illiterates	111 (43.7%)	123 (49.4%)	
**Occupation**	Active	234 (92.5%)	225 (87.5%)	0.06
	Sedentary	19 (7.5%)	32 (12.5%)	
**Smoking**	No	127 (50.3%)	133 (53.4%)	0.49
	Yes	125 (49.6%)	116 (46.6%)	
**Alcohol**	No	235 (93.3%)	245 (94.6%)	0.52
	Yes	17 (6.7%)	14 (5.4%)	

*t- test used for comparison of mean (SD).

### Global DNA methylation and air pollution

**Fig 1:**
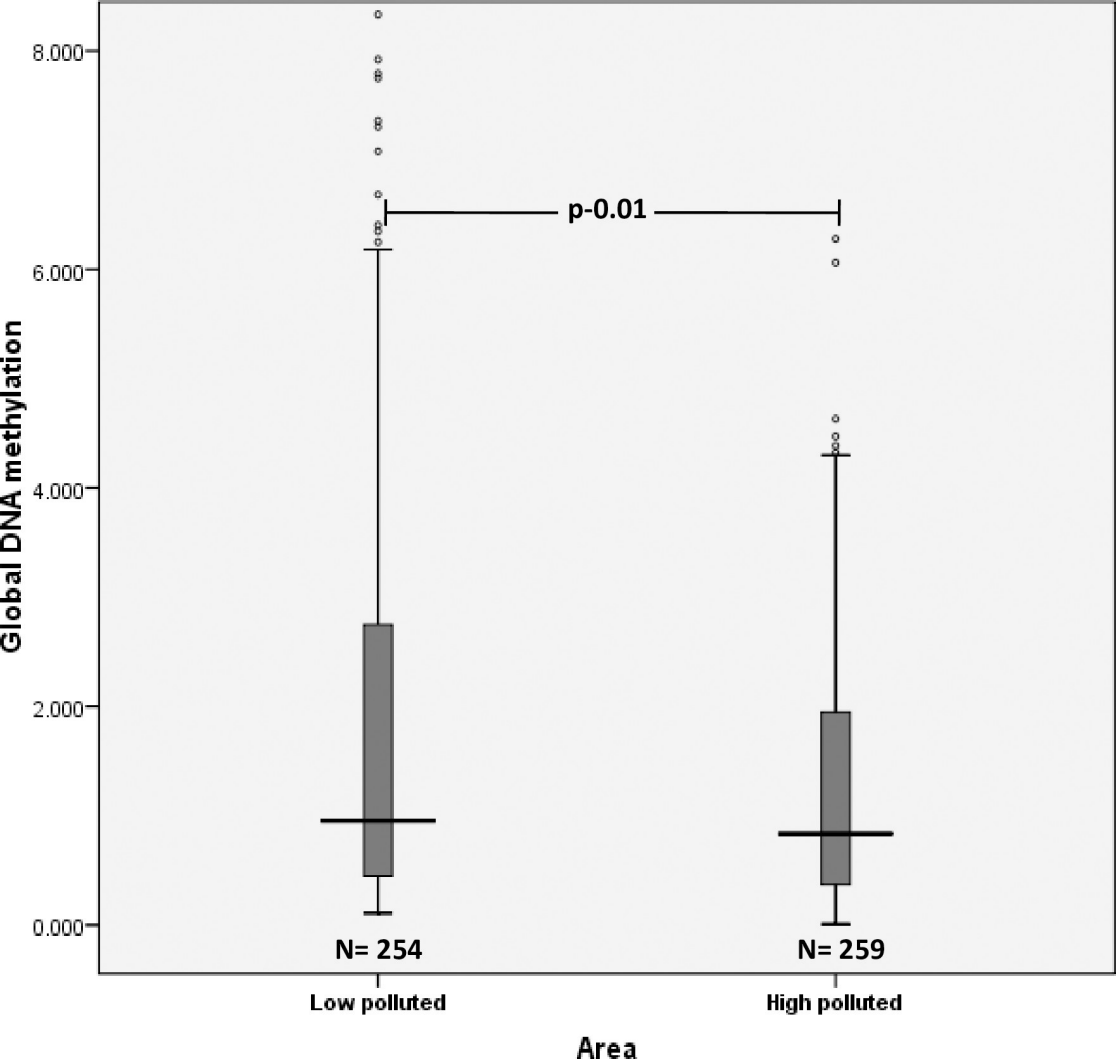
Distribution of median levels of global DNA methylation in low and high polluted areas.

In the overall population, median (IQR) level of global DNA methylation was found to be 0.90 (0.40–2.37). Median levels of global DNA methylation were seen to be significantly reduced in high polluted areas [0.84 (0.37–1.97)] as compared to low polluted areas [0.96 (0.45–2.75)] (p = 0.01, r = -0.383) (Fig 1).

### Air pollution, global DNA methylation and hyperhomocysteinemia

**Fig 2:**
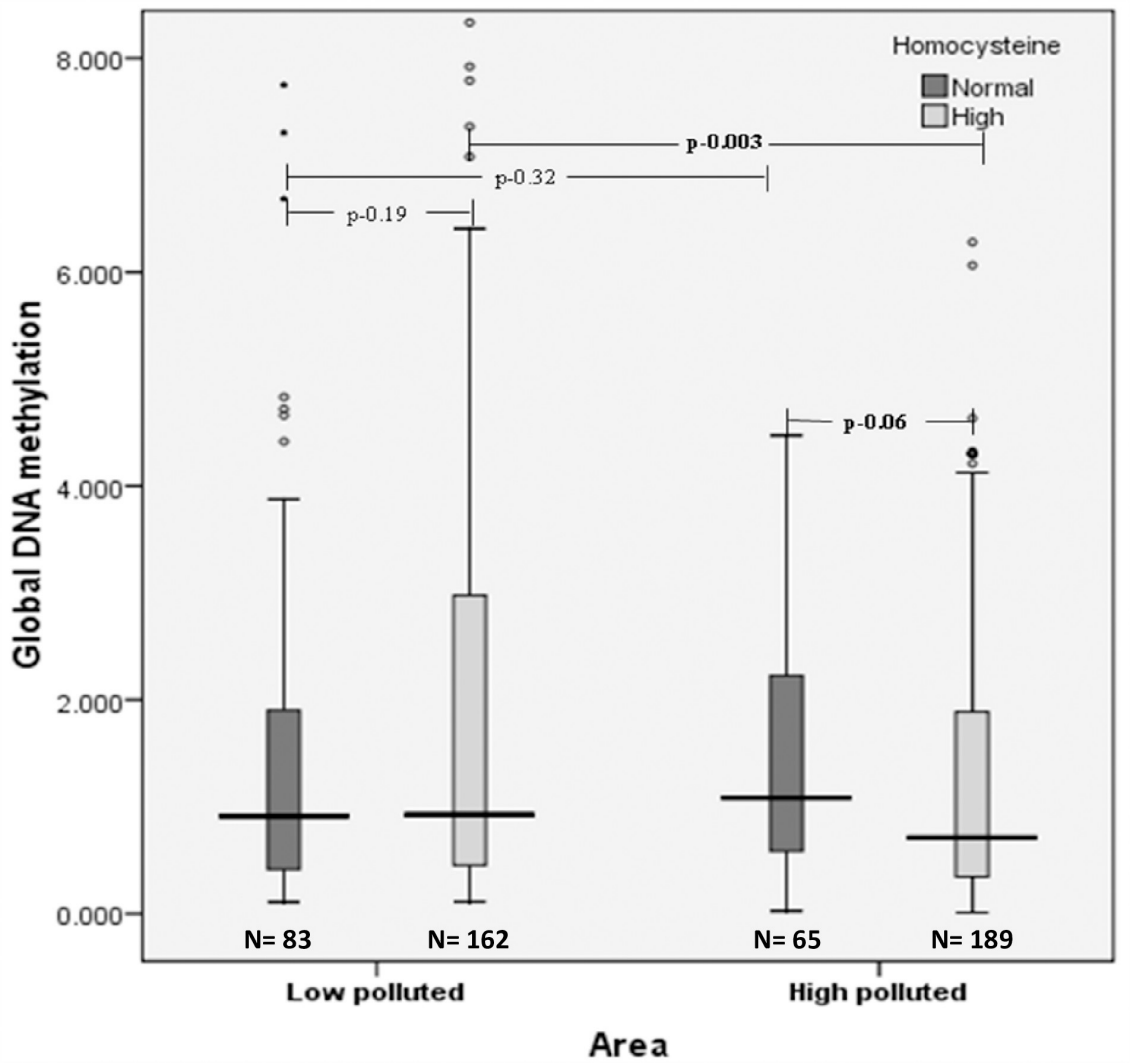
Distribution of median levels of global DNA methylation with respect to high homocysteine in low and high polluted areas.

Global DNA methylation levels were assessed with respect to homocysteine status. In the overall population, global DNA methylation levels were found to be reduced among individuals with hyperhomocysteinemia [0.81 (0.38–2.58)] compared to individuals with normal homocysteine [0.99 (0.43–2.13)], with no statistical significance (p = 0.58) and small effect size (r = -0.02) (Fig 2).

In low polluted areas, global DNA methylation levels were found to be slightly higher [0.93 (0.45–3.00)] among individuals with hyperhomocysteinemia compared to normal homocysteine individuals [0.91 (0.40–1.99)], albeit with no statistical significance (p = 0.19) and small effect size (r = -0.08). However, in high polluted areas, global DNA methylation levels were found to be decreased among individuals with hyperhomocysteinemia [0.71 (0.34–1.90)] compared to individuals with normal homocysteine [1.08 (0.55–2.33)] (suggestive p = 0.06) with moderate effect size (r = -0.313). Further, with respect to hyperhomocystenemia, a significant drop in global DNA methylation was seen among individuals in high polluted areas [0.71 (0.34–1.90)] compared to their respective counterparts in low polluted areas [0.93 (0.45–3.00)] despite multiple corrections (p = 0.003, corrected p = 0.006) and large effect size (r = -0.0512). However, when individuals with normal homocysteine levels in low and high polluted areas were compared, global DNA hypermethylation was seen in high polluted areas [1.08 (0.55–2.33)] compared to low polluted areas [0.91 (0.40–1.99)], with no significant differences despite correcting for multiple comparisons (p = 0.32, corrected p = 0.64) with small effect size (r = 0.08) (Fig 2).

Stepwise regression analysis revealed that air pollution was significantly inversely associated with global DNA methylation among individuals with hyperhomocysteinemia only (β = -0.936, p<0.001) and not among normal homocysteine individuals (β = 0.23, p = 0.81).

### Air pollution, micronutrient deficiencies (folate, vitamin B_12_) and methylation

**Fig 3:**
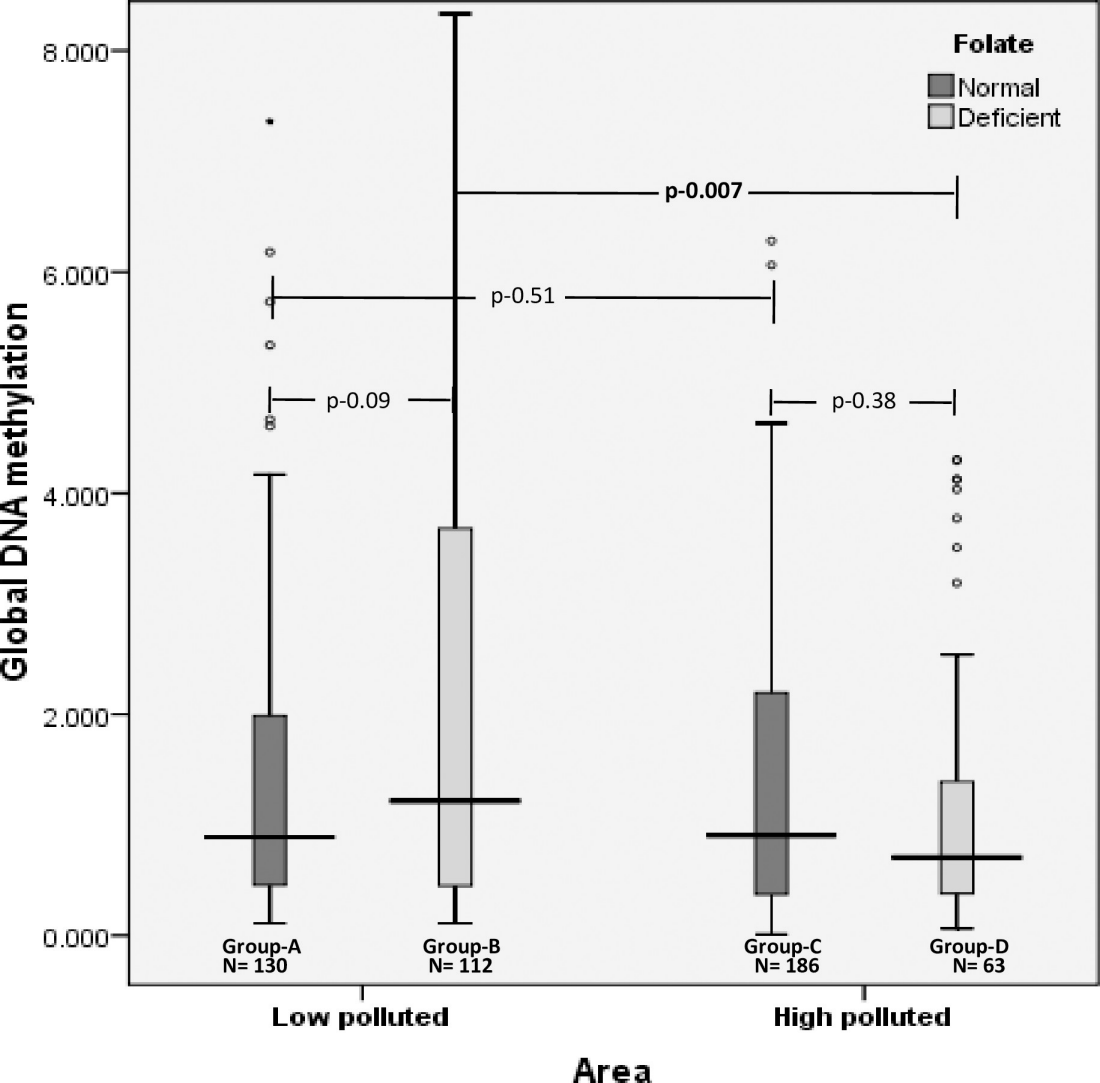
Distribution of median levels of global DNA methylation with respect to folate deficiency in low and high polluted areas.

As one of the reasons for hyperhomocysteinemia is micronutrient deficiencies in form of vitamin B_12_ and folate, the effect of folate and vitamin B_12_ deficiencies on global DNA methylation was seen in low and high polluted areas. With respect to folate deficiency in the overall population, global DNA methylation was seen to be slightly higher among individuals with folate deficiency [0.96 (0.43–2.89)] compared to individuals with normal folate levels [0.90 (0.41–2.15)], with no statistical significance (p = 0.89) and small effect size (r = -0.05) (Fig 3).

Based on the folate levels, individuals in low and high polluted areas were divided into four groups–Group A–normal folate in low polluted, Group B–folate deficient in low polluted, Group C–normal folate in high polluted and Group D–folate deficient in high polluted.

In low polluted areas a similar trend was observed with slightly elevated levels of global DNA methylation in Group B [1.21 (0.45–3.71)] compared to Group A [0.89 (0.46–1.99)], with no statistical significance (p = 0.09) and small effect size (r = 0.107). In contrast, in high polluted areas global DNA hypomethylation was seen in Group D [0.71 (0.38–1.41)] compared to Group C [0.91 (0.37–1.23)] albeit with no statistical significance (p = 0.38) and small effect size (r = 0.054).

With respect to normal folate levels, global DNA methylation was just slightly higher Group C [0.91 (0.36–2.27)] compared to Group A [0.90 (0.45–1.98)], with no statistical difference (p = 0.51, corrected p = 1.02) and with small effect size (r = 0.025). However, global DNA methylation levels were significantly reduced in Group D [0.70 (0.37–1.29)] compared to Group B [1.21 (0.45–3.65)] (p = 0.007, corrected p = 0.01) with large effect size (r = 0.509) (Fig 3).

Further, in the overall population, median (IQR) homocysteine levels were found to be significantly high among individuals with folate deficiency [22.3 (14.3–32.9)] compared to normal folate individuals [19.4 (13.9–27.2)] (p-0.02) (S2 Table). Stepwise linear regression analysis (after controlling for confounder effect including homocysteine) revealed that air pollution was significantly inversely associated with global methylation among individuals with folate deficiency only (β = 1.23, p<0.001) and not among individuals with normal folate levels (β = 0.255, p = 0.22).

**Fig 4:**
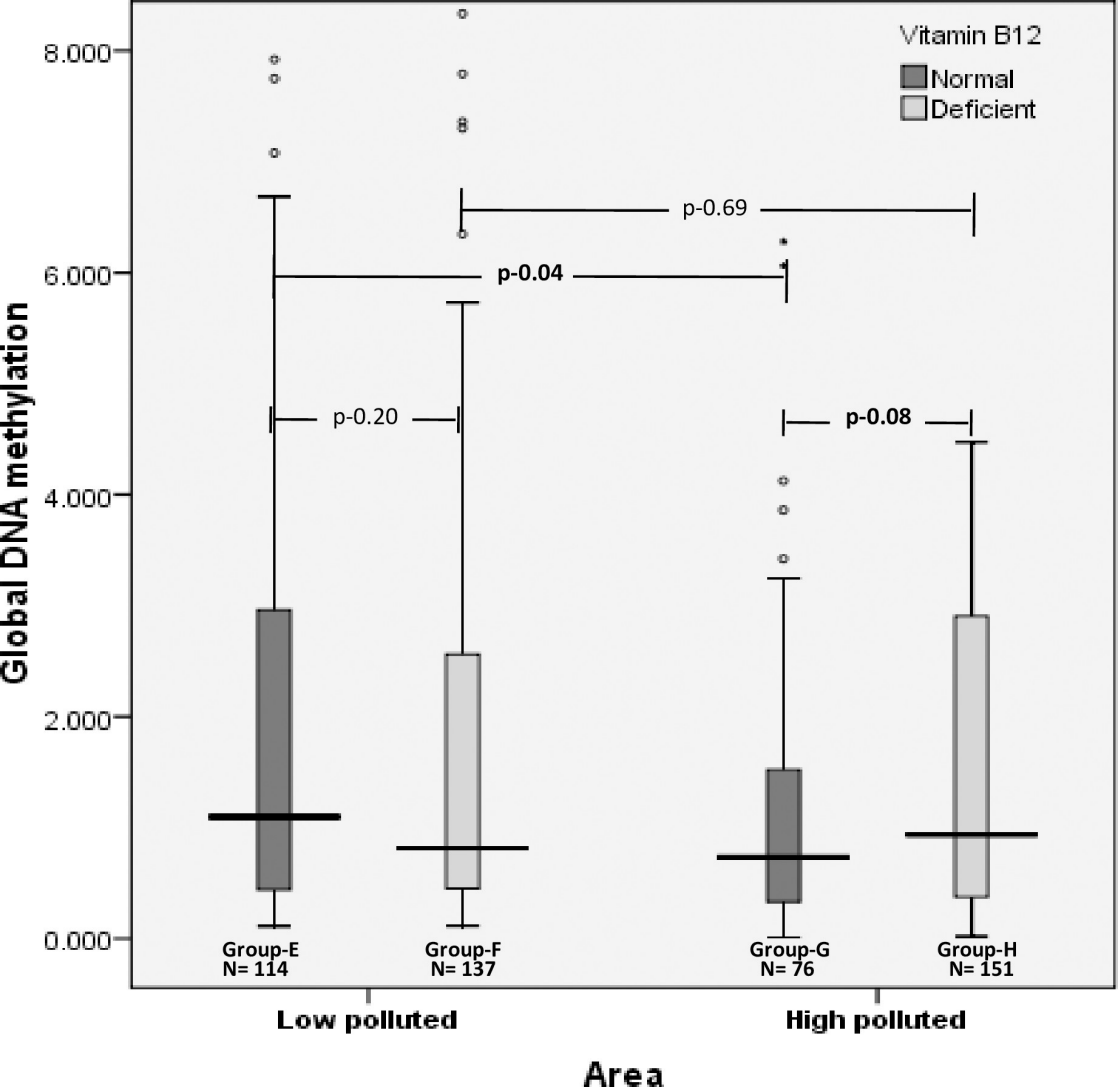
Distribution of median levels of global DNA methylation levels with respect to vitamin B_12_ deficiency in low and high polluted areas.

Further, the global DNA methylation levels were assessed with respect to vitamin B_12_ deficiency. In the overall population, global hypomethylation was seen among individuals with vitamin B_12_ deficiency [0.85 (0.41–2.71)] compared to normal vitamin B_12_ levels [0.96 (0.39–2.17)], with no statistical significance (p = 0.73) and small effect size (r = 0.030) (Fig 4).

Based on the vitamin B_12_ levels, individuals in low and high polluted areas were divided into four groups–Group E–normal vitamin B_12_ in low polluted, Group F–vitamin B_12_ deficient in low polluted, Group G–normal vitamin B_12_ in high polluted and Group H–vitamin B_12_ deficient in high polluted.

In low polluted areas, a similar trend was observed, with reduced levels of global DNA methylation in Group F [0.81 (0.45–2.57)] compared to individuals in Group E [1.10 (0.44–2.99)] (p = 0.20) and small effect size (r = 0.079). However, in high polluted areas, global DNA methylation levels were found to be relatively higher in group H [0.94 (0.37–2.92)] compared to group G [0.74 (0.32–1.54)] (suggestive p = 0.08) with small effect size (r = 302–0.113). Global DNA hypermethylation was observed in Group H [0.94 (0.37–2.91)] compared to individuals in Group F [0.81 304 (0.44–2.57)], albeit with no statistical significance (p = 0.69, corrected p = 1.38) and small effect 305 size (r = 0.022). However, global DNA hypomethylation was seen in Group G [0.73 (0.32–1.53)] compared to Group E [0.81 (0.44–2.57)] with statistically significant differences (p = 0.004, corrected p = 0.08) and moderate effect size (r = 0.307) (Fig 4).

Further, in the overall population, median (IQR) homocysteine levels were found to be significantly high among individuals with vitamin B_12_ deficiency [21.8 (15.3–29.9)] compared to normal vitamin B_12_ individuals [16.10 (11.6–24.7)] (p<0.001) (S2 Table). Stepwise linear regression analysis (after controlling for confounder effect including homocysteine) revealed that air pollution was significantly inversely associated with global methylation among individuals with normal vitamin B_12_ levels (β = 0.23, p<0.001) and not among individuals with vitamin B_12_ deficiency (β = 0.72, p = 0.22).

Additionally, folate deficiency was found to be significantly higher in low polluted areas (p<0.001) while vitamin B_12_ deficiency was significantly higher (p = 0.008) in high polluted areas compared to their respective counterparts (S1 Table). Further, folate deficiency was then seen in light of vitamin B_12_ levels in low and high polluted areas. No significant differences were observed between vitamin B_12_ deficient individuals of low and high polluted areas with respect to folate deficiency (p = 0.09). However, number of individuals with both folate and vitamin B_12_ deficiency were significantly lower in high polluted areas (p<0.001) (S3 Table).

Further, median global DNA methylation levels were seen with respect to micronutrient deficiencies i.e., folate and vitamin B_12_ in low and high polluted areas (Table 2). In low polluted areas, despite folate deficiency, if normal vitamin B_12_ levels were maintained, global DNA methylation levels were found to improve significantly [2.03 (0.60–5.24)] (p = 0.007), whereas in vitamin B_12_ deficient state folate deficiency seemed to play no important role (p = 0.82). Conversely, in high polluted areas when the normal folate status was maintained, global DNA methylation status was seen to significantly improve among vitamin B_12_ deficient individuals [0.91 (0.36–1.63)] compared to vitamin B_12_ normal individuals [0.54 (0.26–1.13)] (p = 0.04). Despite normal vitamin B_12_ levels in high polluted areas, folate deficiency seems to be reducing global DNA methylation levels. Global DNA methylation was also significantly reduced in normal vitamin B_12_ but folate deficient individuals in high polluted areas compared to their respective counterparts in low polluted areas (p_2_ = 0.002) (Table 2).

**Table 2:** Distribution of median (IQR) values of global DNA methylation levels with respect to micronutrient deficiency in low and high polluted areas.

	Low polluted			High polluted		
	Vitamin B_12_ normal	Vitamin B_12_ deficient	p-value	Vitamin B_12_ normal	Vitamin B_12_ deficient	p-value
**Folate normal**	1.00 (0.43–1.78)	0.82 (0.46–2.31)	0.88	0.91 (0.36–1.63)	1.03 (0.37–2.98)	0.04
**Folate deficient**	2.03 (0.60–5.24)	0.81 (0.41–2.89)	0.22	0.54 (0.26–1.13)	0.77 (0.38–2.33)	0.28
**p-value**	0.007	0.82		0.39	0.72	
	p_1_ = 0.30; p_2_ = 0.002; p_3_ = 0.85; p_4_ = 0.59					

p_1_—normal vitamin B_12_ normal Folate low vs high; p_2_—normal vitamin B_12_ deficient Folate low vs high; p_3_—deficient vitamin B_12_ normal Folate low vs high; p_4_—deficient vitamin B_12_ normal Folate low vs high.

### Multiple linear regression analysis for global DNA methylation levels

The collinearity diagnostics (tolerance and VIF) suggested that there was not any obvious multi-collinearity among the selected variables. The minimum eigen value was 0.173 and maximum condition index was 5.427. Multiple regression analysis revealed that air pollution (β = -0.552, p = 0.008) and homocysteine (β = -0.425, p = 0.06) were strong indicators for global DNA methylation after the adjustment of confounders such as age, sex, smoking, alcohol consumption, education and occupation. Air pollution also remained the significant determinant for reduction in global DNA methylation levels in the stepwise backward regression model (Table 3).

**Table 3:** Stepwise multiple linear regression analysis for the effect of independent variables on global methylation.

Variable	β	SE	T	p-value
**Constant**	2.129	0.532	4.000	<0.001
**Age**	-0.011	0.011	-0.993	0.321
**Sex**	0.152	0.203	0.684	0.383
**Smoking**	0.176	0.220	0.798	0.425
**Alcohol**	-0.108	0.401	-0.269	0.788
**Folate**	0.292	0.217	1.347	0.179
**Vitamin B**_**12**_	-0.117	0.215	-0.543	0.587
**Homocysteine**	0.425	0.231	1.84	0.06
**Air pollution**	-0.552	0.209	-2.64	0.008

### Multiple linear regression analysis for global DNA methylation levels in low and high polluted areas

The collinearity diagnostics (tolerance and VIF) suggested that there was not any obvious multi-collinearity among the selected variables in both low and high polluted areas. The minimum eigen value was 0.246 and maximum condition index was 4.19 in low polluted areas. The minimum eigen value was 0.206 and maximum condition index was 4.45 in high polluted areas. Stepwise backward multiple regression analysis revealed that folate deficiency in low polluted areas (β = -0.721, p = 0.02) and vitamin B_12_ deficiency in high polluted areas (β = -0.442, p = 0.03) was significant determinant for global methylation after the adjustment of confounders such as age, sex, smoking, alcohol consumption, education and occupation (Table 4).

**Table 4:** Stepwise backward linear regression analysis for the effect of independent variables on global methylation in low and high polluted areas.

**Low polluted**				
**Variable**	**β**	**SE**	**T**	**p-value**
**Constant**	1.717	0.220	7.817	<0.001
**Folate**	-0.721	0.325	-2.216	0.02
**High polluted**				
**Variable**	**β**	**SE**	**T**	**p-value**
**Constant**	2.116	0.477	4.440	<0.001
**Vitamin B**_**12**_	-0.442	0.208	-2.132	0.03

## Discussion

The present study tried to explore the association of air pollution and homocysteine on global DNA methylation. Global DNA hypomethylation in high polluted areas compared to low polluted areas, as seen in the present study could be a result of air pollution as also supported by other literary evidences where exposure to outdoor air pollution is linked to global DNA hypomethylation [27, 32–34, 42]. A summary of the various cross-sectional and cohort studies conducted across different regions that have explored relation between air pollution and global DNA methylation is provided in Table 5 [27, 34, 36, 43–59]. Majority of the studies used the methylation measurement of repetitive elements/regions (Long interspersed element-1 [LINE-1] or Alu) as a proxy for global DNA methylation assessment. The exact mechanisms have not been illustrated in these studies but a plausible role via inflammation has been proposed in few of these studies (Table 5).

**Table 5:** Studies reporting relation between air pollution and global DNA methylation.

Reference	Country	Study design	Study population	Sample type	Studied region	Methylation status with respect to air pollution
De Prins et al., [27]	Belgium	Prospective cross-sectional	Adults	Whole blood	Global	Hypomethylation associated with NO_2_, PM_10_, PM_2.5_ and O_3_
Madrigano et al., [34]	USA	Cohort	Adults	Whole Blood	Alu, LINE-1	Hypomethylation associated with black carbon in LINE-1 but not Alu, PM_2.5_ not associated with LINE-1 or Alu
Tarantini et al., [36]	Italy	Cohort	Adults	Whole blood	Alu, LINE-1	Hypomethylation associated with PM_10_ in Alu and LINE-1
Bollati et al., [43]	Italy	Cross-sectional	Adults	Whole blood	Alu, LINE-1	Hypomethylation associated with airborne benzene in LINE-1 and Alu
Bacarelli et al., [44]	USA	Cohort	Adults	Blood	Line-1	Hypomethylation associated with short term particulate matter and black carbon exposure in LINE-1
Rusiecki et al., [45]	Denmark	Cross-sectional	Adults	Whole Blood	Alu, LINE-1	Hypomethylation associated with Polychlorinated biphenyls associated in Alu but not LINE-1
Kim et al., [46]	Korea	Cross-sectional	Adults	Whole blood	Alu, LINE-1	Hypomethylation associated with organochlorine pesticides in Alu but not associated with LINE-1 assay
Fustinoni et al., [47]	Italy	Cross-sectional	Adults	Peripehral blood cells	Alu, LINE-1	Global DNA hypomethylation associated with airborne benzene but not with urinary benzene
Duan et al., [48]	China	Cohort	Adults	Whole blood	LINE-1	Hypomethylation associated with polycyclic aromatic hydrocarbons in LINE-1
Kile et al., [49]	USA	Cohort	Adults	Whole Blood	Alu, LINE-1	Global DNA methylation not associated with PM_2.5_ in Alu or LINE-1
Sanchez-Guerra et al., [50]	China	Cross-sectional	Adults	Whole blood	Global DNA methylation	Global DNA methylation not associated with PM_2.5_ or PM_10_
Byun et al., [51]	USA	Cross-sectional	Adults	Whole blood	Blood mtDNA methylation	Hypomethylation associated with PM_2.5_
Chi [52]	USA	Cohort	Adults	Whole blood	Alu, LINE-1	Global DNA methylation (Alu, LINE-1) not associated with long term ambient air pollution levels
Chi et al., [53]	USA	Cross-sectional	Adults	Circulating Monocytes from whole blood	Alu, LINE-1	Global DNA methylation not associated with NO_x_ or PM_2.5_
Plusquin et al., [54]	Netherlands, Italy	Cross-sectional	Adults	Whole blood	Global	Hypomethylation associated with NO_2_ and NO_x_
Lee et al., [55]	Korea	Cross-sectional	Adults	Whole blood	Alu, LINE-1	Hypomethylation associated with sum of persistent organic pollutant levels in men; hypermethylation associated with sum of persistent organic pollutant levels in women
De Nys et al., [56]	Belgium	Cohort	Adults	Buccal cells	Global	Hypomethylation associated with PM_2.5_ and PM_10_
Barchitta et al., [57]	Italy	Cross-sectional	Adults	Whole Blood	LINE-1	Hypomethylation associated with monthly mean PM_10_ levels in LINE-1
Wang et al., [58]	USA	Cross-sectional	Adults	Frozen whole blood	LINE-1	Global DNA methylation (LINE-1) not associated with PM_2.5_
Wang et al., [59]	China	Cross-sectional	Adults	Whole blood	Global	Hypomethylation associated with PM_2.5_ and polycyclic aromatic hydrocarbons

A summary of the results of the present study with respect to methylation, air pollution, homocysteine, folate and methylation levels is presented in Table 6.

**Table 6:** Summary comparison of methylation levels in the present study.

	Overall		Low polluted		High polluted	
**5mC%**			-		**↓↓**	
**Homocysteine**	Normal	High	Normal	High	Normal	High
	-	↓	-	↑	-	↓
**Folate**	Normal	Deficient	Normal	Deficient	Normal	Deficient
	-	↑	-	↑	-	↓
**Vitamin B**_**12**_	Normal	Deficient	Normal	Deficient	Normal	Deficient
	-	↓	-	↓	-	↑
**Normal homocysteine**			↑		-	
**High homocysteine**			-		**↓↓**	
**Normal folate**			↑		-	
**Folate deficient**			-		**↓↓**	
**Normal vitamin B**_**12**_			-		**↓↓**	
**Vitamin B**_**12**_ **deficient**			-		↑	

↑ indicates hypermethylation, ↓ indicates hypomethylation, **↓↓** indicates significant hypomethylation.

In the present study, global DNA hypomethylation was found to be associated with hyperhomocysteinemia and air pollution. Large changes in global DNA methylation levels are also a reflection of hyperhomocysteinemia [60]. The accumulation of homocysteine leading disturbances in one-carbon metabolic pathway which in turn leads to increased intracellular levels of S-adenosylhomocysteine (SAH), a transmethylation inhibitor. The remethylation of homocysteine to methionine is an important step for the systemic supply of methyl donors for global DNA methylation [46]. Thus, in hyperhomocysteinemic conditions, the availability of free methyl groups for DNA methylation is reduced and leads to global DNA hypomethylation.

This effect of hyperhomocysteinemia on global DNA hypomethylation is magnified by air pollution due to which hyperhomocysteinemic individuals in high polluted areas were found to have least global DNA methylation levels. Air pollution induces DNA methylation changes implicated in inflammation and oxidative stress due to long term exposure to particulate emissions [17, 61]. Air pollution also results into elevated homocysteine levels [18] through inflammation and oxidative stress and further cause alteration in the methylation levels [53, 62]. When relation between homocysteine and global DNA methylation is assessed, they seem to show contrary results in high and low polluted areas. When the bad phenotype (i.e., hyperhomocystenemia) is placed in good environment (low pollution), global methylation levels are apparently improved. Whereas, the bad phenotype when placed in bad environment (high pollution), further aggravates reduction in global DNA methylation levels.

DNA methylation is dependent on one-carbon metabolic pathway which in turn relies on methyl nutrients that include B vitamins including folate and vitamin B_12_ [17]. A methyl-nutrient-deficient diet leads to aberrant DNA methylation or global DNA hypomethylation [63]. As expected, folate deficiency was seen to significantly lower global DNA methylation levels in high polluted areas. This could possibly be through pronounced increase in homocysteine levels enhanced by air pollution. No such trend significant trend was observed in methylation levels in terms of vitamin B_12_ deficiency in high and low polluted areas.

Such a result was bit intriguing and the potential reason for this could be looked in light of folate levels. Folate deficiency was found to be least in vitamin B_12_ deficient group in high polluted areas. In other words, in high polluted areas vitamin B_12_ deficiency was seen to be compensated by folate repletion and may improve methylation status. On the contrary, in low polluted areas, the relation between global DNA methylation, vitamin B_12_ and folate seems to be differing where despite folate deficiency global DNA methylation was increased in vitamin B_12_ normal conditions. This hints towards differential mechanisms to manage methylation status under the pressure of air pollution. Improvement in global methylation level among folate deficient but vitamin B_12_ normal individuals in low polluted areas suggests that vitamin B_12_ could be compensating for folate deficiency to maintain global DNA methylation levels in these areas. In contrast, improvement in global DNA methylation among folate normal but vitamin B_12_ deficient individuals in high polluted areas suggest that folate could be compensating for vitamin B_12_ deficiency in high polluted areas. The hypothesis of the present study that air pollution through hyperhomocysteinemia leads to a decline in global DNA methylation may be supported by the findings that it was found to be significantly lower among hyperhomocysteinemic individuals in high polluted areas compared to low polluted areas. In the overall studied population in low and high polluted areas (manuscript communicated elsewhere), hyperhomocysteinemia was found to be significantly higher in high polluted areas, despite significantly lower micronutrient deficiencies (vitamin B_12_ and folate) in low polluted areas. We proposed that the reasons for hyperhomocysteinemia in low and high polluted areas could be different.

Recent evidence suggests that the supplementation of B vitamins attenuate the effect of short term ambient fine particle exposure on methylation by inducing methylation changes in genes involved in mitochondrial oxidative energy metabolism. This acts as an adaptive stress response to eliminate oxidative damage due to exposure to fine particles [17]. However, no evidence is available on the effects of long-term air pollution exposure on methylation in light of B vitamins. In the present study it was found that in order to sustain improvement in global DNA methylation levels, different micronutrients (folate and vitamin B_12_) need to be considered to moderate the effects of air pollution. In high polluted areas, folate repletion could be more beneficial whereas in low polluted areas, vitamin B_12_ repletion could be more functional.

**Fig 5:**
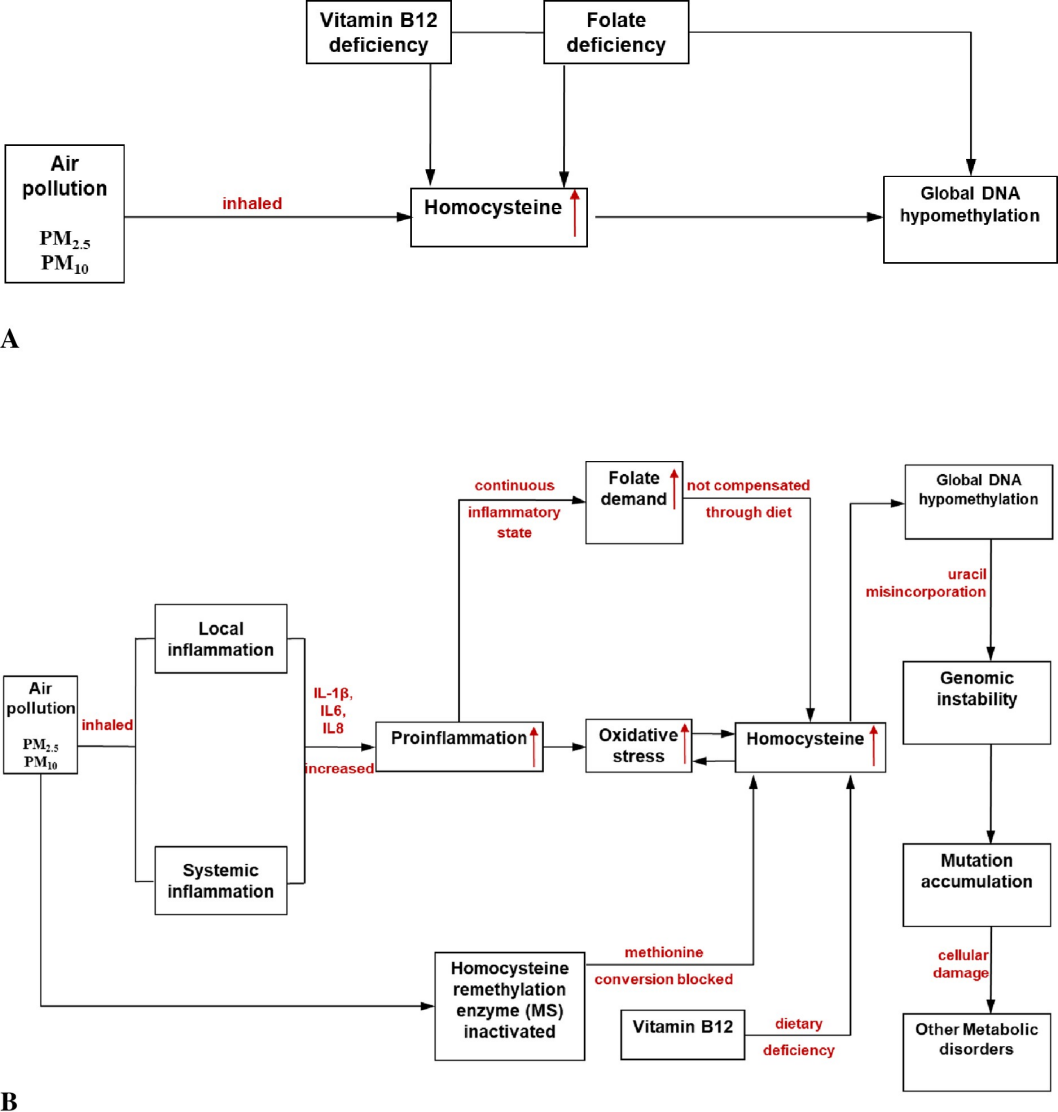
A. The link between air pollution, homocysteine, micronutrient deficiencies and global DNA methylation. Air pollution as well as micronutrient (folate and vitamin B_12_) deficiencies lead to increase in homocysteine levels and further cause global DNA methylation. Both folate and vitamin B_12_ seem to play differential role in the causation of global DNA hypomethylation in the present study. B. The proposed mechanisms for global DNA hypomethylation due to air pollution could be through the key precursor homocysteine. The inhalation of air pollutants (PM_2.5_, PM_10_) triggers local inflammation (in lung epithelia) and systemic inflammation (in blood). This leads to increase in proinflammation due to release of cytokines (IL-1β, IL-6, IL-8 etc.) thereby increasing oxidative stress. Oxidative stress and homocysteine have a cause-effect relation and it results into increased homocysteine. On the other hand, continuous inflammatory state leads to an increased folate demand, which when not compensated through dietary supply leads to homocysteine increase. Dietary deficiency of vitamin B_12_ also leads to homocysteine increase due to disturbances in one-carbon metabolic pathway. Air pollutants (PM_2.5_, PM_10_) also lead to inactivation of homocysteine remethylation enzyme (methionine synthase) due to which conversion of methionine is blocked and ultimately leads to increased homocysteine. Increased homocysteine alters the plasticity of genomic DNA methylation and leads to genomic instability and accumulation of mutations. This further makes an individual prone to other metabolic disorders such as cardiovascular diseases, cancers, neurological problems.

Zhong et al. [17] have suggested that supplementation with B vitamins can attenuate the epigenetic effect of ambient fine air pollutants. This may be the result of lowering of homocysteine due to B vitamin correction through supplementation. The reasons for homocysteine increase could be many, of which micronutrient deficiency (vitamin B_12_ and folate) is one [64]. Oxidative stress, high lipids and air pollution have also been reported to be associated with homocysteine increase [65, 66], though the cause-effect relation of these variables with homocysteine is complex. Still, homocysteine seems to be the central key molecule regulating global DNA methylation status. The results of the present study indicate that in natural settings, B vitamins may play diverse role in different settings to improve/maintain global DNA methylation status as indicated in Fig 5A. Indiscriminate supplementation of B vitamins may cause folate-vitamin B_12_ imbalance or folate trap in the system as dietary form of folate is distinct from the supplemented form [67] which may cause more harm than benefit. Lucock et al. [68] have also pressed upon the calculative health risks of B vitamin supplementation to mitigate cellular effects of air pollution.

The possible mechanisms for aberrant DNA methylation as a result of air pollution have been illustrated in Fig 5B. Long term exposure to environmental air pollutants may lead to local inflammation (in lung epithelia) and systemic inflammation when absorbed [17]. This may create a state of enhanced proinflammation due to increased IL-1*β*, IL-6 and IL-8 cells to counteract the inflammation which in turn may increase oxidative stress in the system. Inflammatory cells (such as IL-1*β*, IL-6, IL-8 and IL-10) liberate reactive species at the site of inflammation and may lead to exaggerated oxidative stress [62]. As a response, these reactive oxygen/nitrogen species may further kick off intracellular signaling gush that may boost proinflammatory gene expression [69, 70]. Thus, inflammation and oxidative stress are suggested to be intimately associated with pathophysiological events that may further lead to an increase in homocysteine levels. A continuous proinflammatory state may also lead to increased folate demand and when this demand is not compensated through dietary supply it may augment homocysteine increase [71]. On the other hand, oxidative stress and hyperhomocysteinemia seem to have a cause-effect relation between them [20, 27, 65, 72, 73]. Dietary deficiency of two vital micronutrients in the homocysteine metabolic pathway i.e. folate and vitamin B_12_ can also lead to hyperhomocysteinemia [64, 74]. Air pollution may augment homocysteine increase by blocking the remethylation pathway where homocysteine is not converted back to methionine due to inactivation of Methionine synthase (MS), a homocysteine remethylation enzyme [20]. Thus, an ultimate increase in homocysteine may consequently lead to global DNA hypomethylation by reducing the supply of free methyl groups in the remethylation pathway [67].

Global DNA hypomethylation may further trigger genomic instability and cellular damage due to uracil misincorporation. It has been suggested that due to the genomic instability, the genome may become more prone to accumulation of mutations [31] which in turn may lead to major metabolic disorders such as cardiovascular diseases, pulmonary diseases etc. [22–26, 74]. Thus air pollution may trigger a cascade of metabolic pathways leading to hyperhomocysteinemia that can cause global DNA hypomethylation [27, 31]. However, the cause-effect relation between air pollution and metabolic adversities is complex and needs to be understood contextually.

## Conclusions

In conclusion, the relation between global DNA methylation and homocysteine is manipulated by external environment (pollution). Air pollution seems to reduce global DNA methylation levels possibly by increasing homocysteine through multiple pathways. The effect on global DNA methylation is intensified when air pollution is accompanied with nutritional deficiencies (vitamin B_12_ and folate) or their consequence i.e. hyperhomocysteinemia. Therefore, individuals in high polluted areas with micronutrient deficiencies may not only be prone to hyperhomocysteinemia but equally open to acquisition of metabolic adversities through global DNA hypomethylation. Further, it is evident that pollution and micronutrient deficiencies may impact methylation levels. It is seemingly difficult to control the macro-level ambient air pollution as it would require policy level interventions. However, the global DNA methylation levels may be improved with dietary modifications and inclusion of vitamin B (folate and B_12_) in the diet at an individual level despite increased air pollution. Along with it, one cannot overlook the differential role of micronutrients in different environmental settings as also seen in the present study. Thus, the supplementation of B vitamins (folate and vitamin B_12_) as suggested by Zhong et al. [17] to attenuate the epigenetic effect of air pollutants needs to be cautiously done. Given the diversity in food habits and cultural practices in different countries, dietary modifications leading to improvement in micronutrient levels need to be considered at population level instead of supplementation, specifically in developing nations like India. Though a major limitation of the present study is the inability to assess the levels of direct exposure to air pollutants but the results indicate a pivotal role of homocysteine as a key precursor for global DNA hypomethylation which is reported to be associated with metabolic health adversities.

## Supporting information

S1 Table Distribution of biochemical variables in low and high polluted areas. (DOC)

S2 Table Distribution of individuals with folate deficiency with respect to vitamin B12 deficiency in low and high polluted areas. (DOCX)

S3 Table Distribution of individuals with folate deficiency with respect to vitamin B_12_ deficiency in low and high polluted areas. (DOCX)

S4 Table Distribution of metabolic adversities in low and high polluted areas. (DOC)
