# Supplementary material for: Association of air pollution and homocysteine with global DNA methylation: A population-based study from North India
Source: PLoS One. 2021 Dec 2;16(12):e0260860. doi: 10.1371/journal.pone.0260860 (PMC8638980; doi:10.1371/journal.pone.0260860)
Supplement: S1 Table — (DOC) [file pone.0260860.s001.doc]

**S1 Table.** Distribution of biochemical variables in low and high polluted areas.

| Variables | Low polluted (N-254) | High polluted (N-259) | Chi square p-value |
| --- | --- | --- | --- |
| *Homocysteine* |  |  |  |
| Normal | 83 (33.9%) | 65 (25.6%) | 0.04 |
| High | 162 (66.1%) | 189 (74.4%) |  |
| *Folate* |  |  |  |
| Normal | 130 (53.7%) | 186 (74.7%) | <0.001 |
| Deficient | 112 (46.3%) | 63 (25.3%) |  |
| *Vitamin B-12* |  |  |  |
| Normal | 114 (45.4%) | 76 (33.5%) | 0.008 |
| Deficient | 137 (54.6%) | 151 (66.5%) |  |
| *Homocysteine*  Median (IQR)** | 18.75 (13.10-27.60) | 20.70 (14.60 - 29.05) | 0.08 |
| *Folate*  Median (IQR)** | 3.46 (2.47- 5.06) | 4.09 (3.06-6.30) | 0.001 |
| *Vitamin B12*  Median (IQR)** | 251.0 (189.25 - 354.0) | 232.0 (188.75 - 326.0) | 0.26 |
