# Supplementary material for: Association of air pollution and homocysteine with global DNA methylation: A population-based study from North India
Source: PLoS One. 2021 Dec 2;16(12):e0260860. doi: 10.1371/journal.pone.0260860 (PMC8638980; doi:10.1371/journal.pone.0260860)
Supplement: S2 Table — (DOCX) [file pone.0260860.s002.docx]

**S2 Table.** Distribution of median (IQR) levels of homocysteine with respect to folate deficiency and vitamin B-12 deficiency in overall population

|  | **Folate Normal** | **Folate Deficient** | **p-value*** |
| --- | --- | --- | --- |
| *Homocysteine*  Median (IQR) | 19.40 (13.90 – 27.2) | 23.88 (14.3 – 32.9) | 0.02 |
|  | **Vitamin B-12 Normal** | **Vitamin B-12 Deficient** |  |
| *Homocysteine*  Median (IQR) | 16.1 (11.65-24.75) | 21.85 (15.3 – 29.95) | <0.001 |

*Mann-Whitney test used for comparison
