# Supplementary material for: Association of air pollution and homocysteine with global DNA methylation: A population-based study from North India
Source: PLoS One. 2021 Dec 2;16(12):e0260860. doi: 10.1371/journal.pone.0260860 (PMC8638980; doi:10.1371/journal.pone.0260860)
Supplement: S3 Table — (DOCX) [file pone.0260860.s003.docx]

**S3 Table.** Distribution of individuals with folate deficiency with respect to vitamin B_12_ deficiency in low and high polluted areas

| Folate Deficiency | **Low polluted** | | **High polluted** | | **χ2 p-value** |
| --- | --- | --- | --- | --- | --- |
|  | **B_12_ normal** | **B_12_ deficient** | **B_12_ normal** | **B_12_ deficient** | p_1_-0.17  p_2_-0.18  p_3_-0.09  p_4_-<0.001 |
|  | 40.7% | 49.6% | 28.8% | 20.7% |  |

p_1_-low polluted B_12_ normal vs deficient; p_2_-high polluted B_12_ normal vs deficient; p_3_- B_12_ normal low polluted vs high polluted; p_4_- B_12_ deficient low polluted vs high polluted
