# Supplementary material for: Association of air pollution and homocysteine with global DNA methylation: A population-based study from North India
Source: PLoS One. 2021 Dec 2;16(12):e0260860. doi: 10.1371/journal.pone.0260860 (PMC8638980; doi:10.1371/journal.pone.0260860)
Supplement: S4 Table — (DOC) [file pone.0260860.s004.doc]

**S4 Table.** Distribution of metabolic adversities in low and high polluted areas.

|  | Low polluted | High polluted | p-value |
| --- | --- | --- | --- |
| Obesity (BMI ≥25kg/m^2^) | 16.1% | 29.6% | 0.003 |
| Hypertension Stage -2  (Systolic blood pressure >140 mmHg; Diastolic blood pressure >90 mmHg) | 49.2% | 45% | 0.05 |
| Metabolic Syndrome * | 28% | 32% | 0.36 |

*metabolic syndrome classified on basis of NCEP-ATP III criteria
